# Epigenetics and Cell Death: DNA Hypermethylation in Programmed Retinal Cell Death

**DOI:** 10.1371/journal.pone.0079140

**Published:** 2013-11-11

**Authors:** Karl J. Wahlin, Raymond A. Enke, John A. Fuller, Giedrius Kalesnykas, Donald J. Zack, Shannath L. Merbs

**Affiliations:** 1 Department of Ophthalmology, Johns Hopkins University School of Medicine, Baltimore, Maryland, United States of America; 2 Department of Neuroscience, Johns Hopkins University School of Medicine, Baltimore, Maryland, United States of America; 3 Department of Molecular Biology and Genetics, Johns Hopkins University School of Medicine, Baltimore, Maryland, United States of America; 4 Institute of Genetic Medicine, Johns Hopkins University School of Medicine, Baltimore, Maryland, United States of America; 5 Department of Ophthalmology, Clinical Research Unit, School of Medicine, University of Eastern Finland, Kuopio, Finland; 6 Institute de la Vision, Université Pierre et Marie Curie, Paris, France; 7 Department of Biology, James Madison University, Harrisonburg, Virginia, United States of America; University of Florida, United States of America

## Abstract

**Background:**

Vertebrate genomes undergo epigenetic reprogramming during development and disease. Emerging evidence suggests that DNA methylation plays a key role in cell fate determination in the retina. Despite extensive studies of the programmed cell death that occurs during retinal development and degeneration, little is known about how DNA methylation might regulate neuronal cell death in the retina.

**Methods:**

The developing chicken retina and the *rd1* and rhodopsin-GFP mouse models of retinal degeneration were used to investigate programmed cell death during retinal development and degeneration. Changes in DNA methylation were determined by immunohistochemistry using antibodies against 5-methylcytosine (5mC) and 5-hydroxymethylcytosine (5hmC).

**Results:**

Punctate patterns of hypermethylation paralleled patterns of caspase3-dependent apoptotic cell death previously reported to occur during development in the chicken retina. Degenerating *rd1* mouse retinas, at time points corresponding to the peak of rod cell death, showed elevated signals for 5mC and 5hmC in photoreceptors throughout the retina, with the most intense staining observed in the peripheral retina. Hypermethylation of photoreceptors in *rd1* mice was associated with TUNEL and PAR staining and appeared to be cCaspase3-independent. After peak rod degeneration, during the period of cone death, occasional hypermethylation was observed in the outer nuclear layer.

**Conclusion:**

The finding that cell-specific increases of 5mC and 5hmC immunostaining are associated with the death of retinal neurons during both development and degeneration suggests that changes in DNA methylation may play a role in modulating gene expression during the process of retinal degeneration. During retinal development, hypermethylation of retinal neurons associates with classical caspase-dependent apoptosis as well as caspase-3 independent cell death, while hypermethylation in the *rd1* mouse photoreceptors is primarily associated with caspase-3 independent programmed cell death. These findings suggest a previously unrecognized role for epigenetic mechanisms in the onset and/or progression of programed cell death in the retina.

## Introduction

Epigenetic modifications to genomic DNA and associated histone proteins dictate chromatin structure and regulate gene expression across a range of cellular processes [1]. DNA methylation is established and maintained in the genome by structurally distinct family members of DNA methyltransferase (Dnmt) enzymes [2]. Dnmts transfer a methyl group from S-adenosyl methionine to a cytosine nucleotide, resulting in a 5-methyl cytosine (5mC) base. The recent discovery that 5mC can be further modified to 5-hydroxymethylcytosine (5hmC), 5-formylcytosine (5fC) and carboxylcytosine (5caC) through the activity of the Tet (ten eleven translocation) proteins increases the complexity by which epigenetically modified cytosine bases can participate in gene regulation [3], [4]. Genome-wide profiles in plants and vertebrates have demonstrated an inverse correlation between transcriptional activity and the accumulation of 5mC in upstream regulatory regions of genes [5], [6]. In contrast, emerging evidence demonstrates a positive correlation between transcription and 5hmC in upstream regulatory regions of genes [7]. 5hmC accumulation has been shown to coincide with depletion of 5mC [8], adding to the evidence that 5mC and 5hmC have reciprocal roles in the dynamic regulation of DNA methylation.

In the retina, cone- and rod-specific genes demonstrate cell-specific patterns of DNA methylation [9], which appear to play an important role in the establishment and/or maintenance of retinal cell type-restricted gene expression. The cell-specific DNA methylation patterns in mature retinal neurons suggest a requirement for both active methylation and demethylation processes in the establishment of differential methylation patterns during retinal development. Abnormal development of photoreceptors (PR) and dysregulation of retinal gene expression is observed with knockdown of *Dnmt1* [10], [11].

Programmed cell death (PCD) plays an important role in retinal development. Inner retinal cells are selectively lost during eye morphogenesis in many species, including mouse and chicken [12]–[14]. However, developmental cell death is not universal among cell populations as some inner retinal neurons, such as amacrine and ganglion cells, are selectively lost while others, such as PRs, are not [12]. In the inner retina of many species, including the chicken, this programmed cell death appears to be caspase-dependent with overlapping TUNEL and caspase signals. Moreover, pharmacological blockade of caspase during development results in increased numbers of retinal ganglion cells [15].

Loss of PRs is a hallmark of the retinal dystrophies, including retinitis pigmentosa (RP). In these disorders, PRs undergo a directed process of cell death ultimately leading to vision loss and often blindness. Numerous animal models recapitulate photoreceptor cell loss and are useful systems in which to explore the pathophysiology of retinal degeneration. The retinal degeneration 1 (*rd1*) mouse is a well-studied animal model of RP. These mice undergo an apoptotic-like PR loss beginning at postnatal day 10 (P10) and peaking at P15. By P21, essentially all rods are lost, with only 1–2 rows of cones remaining [16], [17]. Several recent studies focusing on cell loss in the *rd1* mouse suggest that in this model rods die by a caspase-independent mechanism [18]–[22].

Despite many studies of PCD in models of retinal development and degeneration, little is known about epigenetic changes that occur before, during and after cell death. To explore the role of epigenetic mechanisms during PCD, we used immunohistochemistry to investigate cellular patterns of DNA methylation and hydroxymethylation in the developing chicken retina as well as in mouse models of retinal degeneration. Through these studies, we provide evidence that DNA methylation and hydroxymethylation are linked to PCD during normal development and pathogenic retinal degeneration.

## Materials and Methods

### Animals

All animal experiments were conducted with the approval of the Johns Hopkins Animal Care and Use Committee and the ARVO Statement for the Use of Animals in Ophthalmic and Vision Research. All mice were euthanized using IsoSol™ (VEDCO) exposure followed by cervical dislocation. Retinas from wild-type (*+/+*) and *rd1* (*rd/rd*) mice, each with a C57BL/6J background, were harvested daily from P7–14 during the peak rod death, and at P35 when only cones remain in the outer nuclear layer (ONL). Homozygous rhodopsin-GFP (*hrhoG/hrhoG*) mice, generated by knock-in of a human rhodopsin-EGFP fusion gene at the rhodopsin locus and which demonstrate photoreceptor degeneration, were processed at P24 during a period of early degeneration [23], [24]. Adult C57BL/6J mice were used for retinal explant cultures. Chick embryos (B&E eggs; York Springs, PA) were euthanized by decapitation. Embryonic chicken retinas were harvested at embryonic days 3 (E3) 4, 7, 11, 13, 15 and 20. At E3 and 4, samples were collected at Hamburger and Hamilton stages 21 and 25, respectively [25].

### Tissue Processing and Immunohistochemistry

Tissue processing was performed as described [26], [27]. Enucleated eyes were opened along the ora serrata, vitreous removed and eyecups fixed for 25 min in cold 4% paraformaldehyde in 0.1 M phosphate buffer, pH 7.4. Eyecups were briefly immersed in 6.75, 12.5% sucrose in phosphate buffer, incubated in 25% sucrose in phosphate buffer overnight and in a 2∶1 ratio of 25% sucrose-0.1 M phosphate buffer and OCT Tissue-Tek (Ted Pella; Redding, CA) for 1 hr, and snap-frozen on dry ice/isopentane. Tissue sections cut on a cryostat at 8–10 µm were thaw-mounted onto Superfrost Plus glass slides (Fisher; Pittsburg, PA), blocked and permeabilized in 2–10% normal horse serum in PBS containing 0.1% Triton X-100 and incubated overnight in primary antibody in 2% serum containing 0.1% Triton X-100. Primary antibody sources and dilutions are listed in Table 1. Secondary antibodies were anti-mouse, -sheep and -rabbit IgG’s (H+L) coupled to Alexafluor488, 546, 594 or 647 (Invitrogen, 1∶1,000). 10 µg/ml Hoechst 3342 (Molecular Probes) was used to visualize cell nuclei. Serial sections processed similarly but without primary antibody were used to control for non-specific background.

**Table 1:** Information on primary antibodies used in this study (See also Materials and Methods).

Antibody	Immunogen	Source	Host	Dilution
5hmC	Raised against 5-hydroxymethylcytidine conjugated to KLH	Active motif#39769	R	1∶2,000–1∶10,000
5mC	Sheep were immunized with 5-methylcytosine coupled tokeyhole limpet hemocyanin (KLH)	Life Span;#LSC64477/18463	S	1∶1,000–5,000
5mC (33D3)	Mouse monoclonal antibody (33 D3) was made against5-methylcytosine conjugated to ovalbumin	Genway Biotech;#20-003-40005	M	1∶2,000
5caC	5-Carboxylcytosine antibody was raised against 5-carboxylcytidineconjugated to KLH and recognizes 5-carboxylcytosine	Active motif# 61230	R	1∶1,00–1,1,000
5fC	5-Formylcytosine antibody was raised against 5-formylcytidineconjugated to KLH and recognizes 5-formylcytosine	Active motif#61228	R	1∶100–1∶1,000
Caspase-3 (cleaved)	Amino terminal residues adjacent to (Asp175) in human caspase	Cell SignalingTechnology; #9661	R	1∶500
Caspase-3 (cleaved)	KLH coupled synthetic peptide CRGTELDCGIETD correspondingto amino acids 163–175 of human Caspase 3	R&D system; #AF835	R	1∶500
PAR	Poly(ADP-ribose) polymer	Trevigen#4336-APC-050	R	1∶500
PAR, mAb (10H)	Purified poly(ADP-ribose)	EnzoALX-804-220-R100	M	1∶500

M = mouse monoclonal; R = rabbit polyclonal; S = sheep polyclonal.

### Antibody Characterization

Antibodies used in this study (Table 1) were produced and validated in the following way.

#### A) 5-hydroxymethylcytosine (5hmC)

A rabbit polyclonal antibody (Active Motif, #39769) was made by immunization with 5hmC conjugated to keyhole limpet hemocyanin (KLH). Whole serum 5hmC antibodies tested by dot blot analysis showed light cross reactivity with 5mC labeled oligonucleotides only at concentrations that were substantially higher than those used for IHC (Figure S1B) and through an *in situ* competition assay in which pre-adsorption of 5hmC antibodies with 5hmC labeled PCR products, but not 5mC, effectively quenched antibody signals in cryopreserved tissue section PR’s of P11 degenerating retinas (Figure S1D).

#### B) 5-methylcytosine (5mC)

A mouse anti-5mC IgG antibody conjugated to ovalbumin was purified by protein A chromatography (Genway Biotech, # 20-003-40005). A sheep polyclonal anti-5mC antibody, produced by immunizing with 5-methylcytosine coupled to KLH, was purified by protein-G column chromatography (Life Span, #LSC64477/18463). Specificity of these antibodies was confirmed by dot blot analysis and through a competition assay (Figure S1C, E, F). For the chicken immunohistochemistry, the mouse monoclonal was used since it generally gave a higher detection signal; however, similar patterns were also evident with the sheep antibody (data not shown). For the mouse immunohistochemisty, both antibodies against 5mC gave similar results except that the mouse monoclonal antibody cross-reacted with inner retinal vasculature of mouse sections (data not shown), presumably due to endogenously expressed IgG in those structures.

#### C) Cleaved Caspase-3

Presence of the active form of caspase, cleaved caspase-3 (cCaspase-3), in the ONL of the *rd1* mouse retina during the peak of degeneration has been previously described [21]. Two rabbit polyclonal antibodies were used to detect cCaspase-3. The first antibody (Cell Signaling; #9661) was produced by immunizing animals with a KLH-coupled synthetic peptide corresponding to residues adjacent to Asp175 in human caspase-3 and purified by protein-A and peptide affinity chromatography. Specificity and sensitivity were shown by Western blot of extracts from a mouse cortical ischemic model undergoing apoptotic cell death [28]. Use of this antibody for immunohistochemistry has been previously described in rodents [29] and chicken [30]. The second rabbit polyclonal (R&D systems; #AF835) was produced by immunization with a synthetic KLH coupled peptide CRGTELDCGIETD corresponding to amino acids 163–175 of human caspase-3 and affinity purified. Western blots of recombinant caspase-3 detected the p17 subunit of cCaspase-3 and not its precursor [31], [32]. Specificity of this antibody in a chicken axotomomy model has also been demonstrated [33].

#### (D) Poly(ADP)-Ribosylated (PAR) protein

A rabbit affinity purified IgG polyclonal antibody and a mouse IgG3 monoclonal antibody were each raised against poly(ADP-ribose) (PAR) polymer. Both antibodies used in this study gave similar staining patterns. The accumulation of PAR proteins (PAR) in the ONL of degenerating rodent retinas, including the *rd1* mouse during the peak of degeneration, is well established [34], [35]. Antibody specificity has been verified by western blot [36], [37]. Both antibodies used in this study resulted in preferential staining of photoreceptors undergoing cell death and not in wild-type controls.

### 5mC and 5hmC Dot Blots

5mC and 5hmC modified DNAs were prepared by PCR amplification of a 450bp amplicon from the mouse wild-type ROSA26 locus using cytosine (C), 5-methyl-cytosine (5mC), or 5-hydroxymethyl-cytosine (5hmC) containing dNTP mixes. Primer sequences were R26F 5′-CCC TCG TGA TCT GCA ACT CCA GTC -3′ and R26R 5′AAA GTC GCT CTG AGT TGT TAT-3′. Incorporation of the modified nucleotides into the PCR product was confirmed by digestion with *Bso*BI, a methylation-sensitive restriction enzyme (Figure S1A). DNAs were spotted onto positively charged nylon membranes (GE Healthcare) and UV crosslinked for 1 minute. Membranes were blocked in 5% milk in PBS with 0.05% TWEEN 20 (0.05% PBT) for 1 hour at room temperature. Mouse anti-5mC (Genway; 1∶2,000) and rabbit anti-5hmC (Active Motif, 1∶5,000) antibodies were diluted in 5% milk buffer and mixed in 0.05% PBT and incubated overnight at 4°C. Membranes were washed 3 times in 0.05% PBT, 10 minutes each. Anti-mouse and anti-rabbit HRP-conjugated secondary antibodies (Cell Signaling Inc; 1∶20,000) were diluted in 5% milk buffer mixed in 0.05% PBT and incubated for 30 minutes at room temperature. Membranes were washed 3X in 0.05% PBT, 10 minutes each, incubated with 5 mL of SuperSignal West Pico Chemiluminescent Substrate mix (Thermo Fisher Scientific; Rockford IL) for 5 minutes and detected using a Kodak 4000mm Imaging Station.

### DNA Nick-end Labeling by the TUNEL Method

Tissue sections were processed as above and the TUNEL technique performed as described with minor modifications [38]. In short, tissues were simultaneously permeabilized and blocked with 0.25% Triton-X in PBS containing 2% normal horse serum for 15 minutes, washed in ddH2O and incubated for 30 minutes at 37°C in TUNEL reaction mix comprised of 45 microliters FITC-conjugated TUNEL label mix (200mM potassium cacodylate, 25 mM Tris-HCl, 1 mM CoCl2, 0.25 mg/ml bovine serum albumen, pH 6.6) and 5 µl of TUNEL enzyme (Roche cat# 11767291910; Indianapolis, IN).

### *In vitro* Adult Retinal Explants

Retinal eyecups were gently separated from the sclera and RPE in Ca++ and Mg++free Hanks Buffered Salt Solution (CMF-HBSS) using fine tipped micro-dissection forceps (#55 Dumont). To minimize cell trauma, eyecups were carefully transferred with a wide-bore P1000 pipette tip and flat mounted onto hydrophilic PTFE millicell inserts (cat# PICM01250; 12mm diameter, 0.4 µm pore) for organotypic culture (Millipore; Billerica, MA). Eyecups retaining a small amount of RPE were placed on Millicell-CM membranes with the RPE facing the membrane. These were maintained at 37°C for up to 9 days in Neurobasal medium containing 2% B27 supplement (Invitrogen) with a complete medium exchange performed every 2 days. Explant cultures were fixed in paraformaldehyde, cryoprotected in sucrose and processed for tissue sectioning as detailed above.

### Microscopy and Image Processing

**Figure 1:**
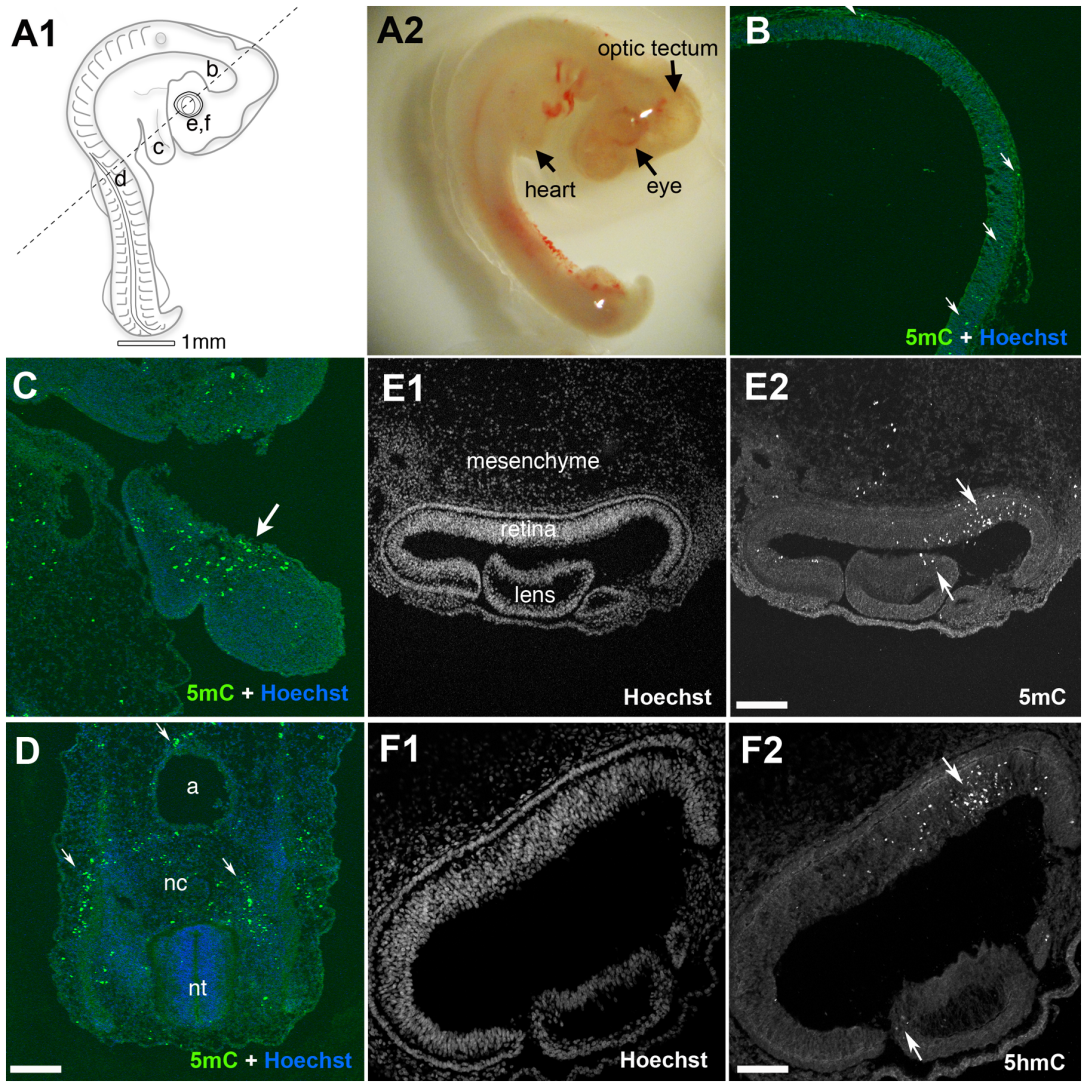
5mC accumulation in the early developing chicken embryo. (A1) A diagram indicating the relative cross sectioned region of tissues studied (lowercase letters correspond to the respective panels below). (A2) A representative embryo at E3.5. (B) 5mC deposits in neurons lining the midbrain optic tectum and (C) a region corresponding to the developing heart. (D) The region adjacent to the neural tube (nt), notochord (nc) and aorta (a). (E1 & F1) Hoechst nuclear counterstained retinal sections correspond to sections in E2 and F2, respectively. (E2) 5mC in the developing retina, lens and surrounding mesenchyme. (F2) 5hmC in the developing retina. Arrows indicate 5mC (+) and 5hmC (+) cells. Scale bars = 100 µM in D, 75 µM in E2 and 50 µM in F2.

**Figure 2:**
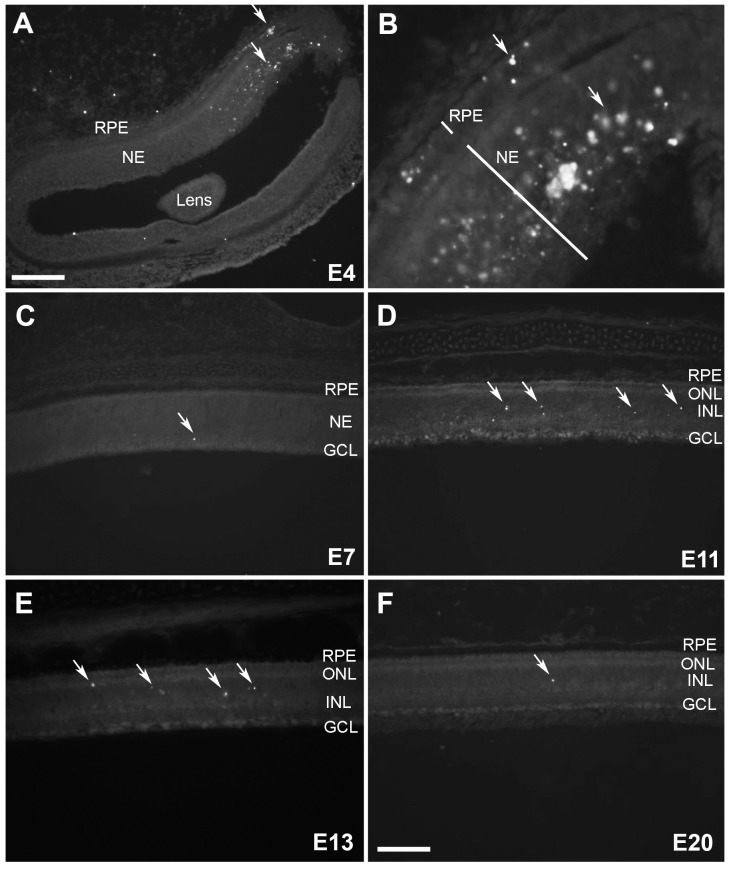
5mC (+) cells in the developing chicken retina. (A) The morphogenic furrow at E4 with a dense patch of 5mC staining (see arrows) (B) A magnified image of 5mC staining in the morphogenic furrow in a serial section taken at higher magnification. (C) 5mC (+) cells at E7, (D) E11, (E) E13 and (F) E20 are indicated by arrows when present. RPE = retinal pigment epithelium; NE = neuroepithelium; ONL = outer nuclear layer; INL = inner nuclear layer; GCL = ganglion cell layer. Scale bars = 75 µM in A and 200 µM in C–F.

**Figure 3:**
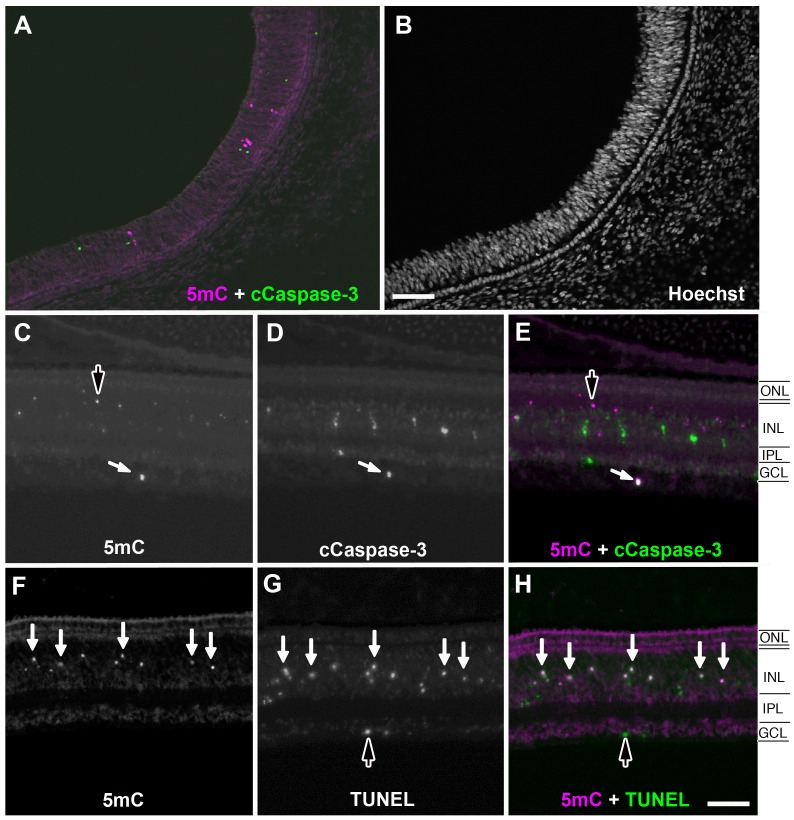
Comparison of cCaspase3 and TUNEL co-labeling with 5mC in the developing chicken retina. (A) Low magnification of 5mC and cCaspase3 staining at E11. (B) Nuclear counterstaining of a low magnification section. (C) 5mC and (D) cCaspase3 staining. Colocalization of 5mC (+) and cCaspase3 (+) cells indicated by white arrows overlaid in (E). Dark arrows indicate 5mC (+) cells that are cCaspase3 (−). (F) 5mC and (G) TUNEL staining at E11. (H) Co-localization indicated by white arrows. TUNEL (+) but 5mC (−) indicated by a dark arrow. ONL = outer nuclear layer; INL = inner nuclear layer; IPL = inner plexiform layer; GCL = ganglion cell layer. Scale bars = 100 µM in A–B; 75 µM in C–H.

**Figure 4:**
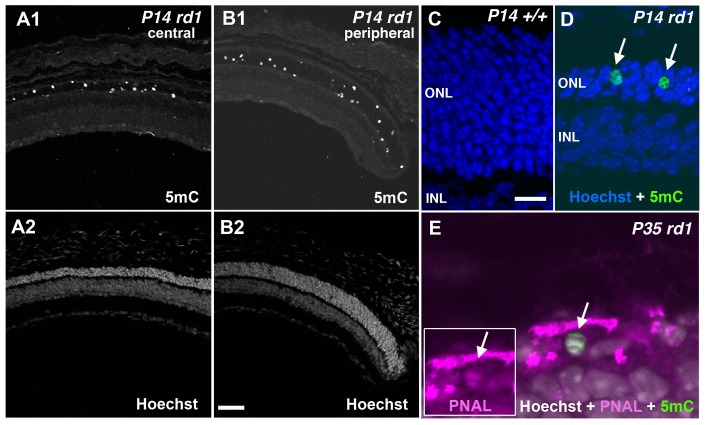
5mC expression in the ONL of the *rd1* mouse retinal degeneration model. (A1, B1) 5mC labeling and (A2, B2) Hoechst labeling of the central and peripheral retina, respectively, of an *rd1* mouse at P14 during the peak of rod degeneration. (C) Age-matched retinas from wild-type control and (D) *rd1* mice, respectively, at P14 with Hoechst (blue) and 5mC (green) staining (arrows). (E) 5mC (green) and cone marker PNAL (magenta) staining co-localize (arrows) in a subset of cones in the remnants of the ONL. ONL = outer nuclear layer; INL = inner nuclear layer. Scale bars = 100 µM (B2) and 35 µM (C).

**Figure 5:**
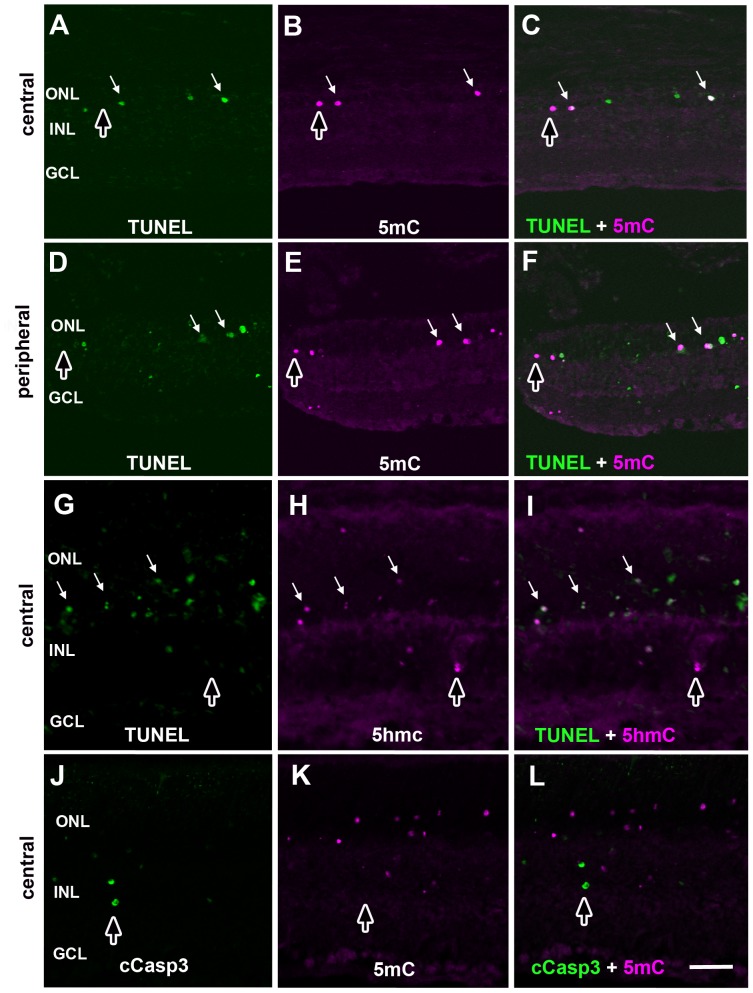
Cell death markers double labeled with 5mC (+) or 5hmC (+) photoreceptors in *rd1* mouse retinas. Retinas from P14 *rd1* mice were double-labeled with TUNEL stain (A, D and G) and 5mC (B and E) or 5hmC (H) with central (A–C, G–I) and peripheral retina (D–F) shown accordingly. Discrete signals for cCaspase3 (J) and 5mC (K) labeling show virtually no overlap in signals (L). Overlap in signals are indicated by white arrows as shown in panels C, F and I. Cells labeled with one marker but not the other are indicated with dark vertical arrows. ONL = outer nuclear layer; INL = inner nuclear layer; GCL = ganglion cell layer. Scale bars = 50 µM.

**Figure 6:**
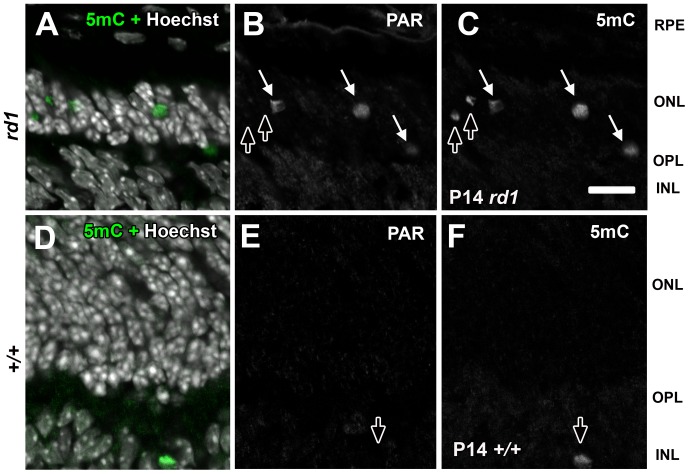
Detection of 5mC and PAR in the outer nuclear layer of degenerating *rd1* retinas. (A–C) P14 *rd1* or (D–F) wild type mouse retinas were labeled with antibodies against 5mC and PAR. Panels A and D are co-labeled with Hoechst nuclear counterstain to indicate the respective retinal layer where 5mC exists. White arrows (B–C, E–F) indicate cells in which both antigens were detected. Dark arrows indicate cells that were labeled with 5mC but not PAR. RPE = retinal pigment epithelium, ONL = outer nuclear layer; OPL = outer plexiform layer, INL = inner nuclear layer; GCL = ganglion cell. Scale bar (L) = 50 µM.

**Figure 7:**
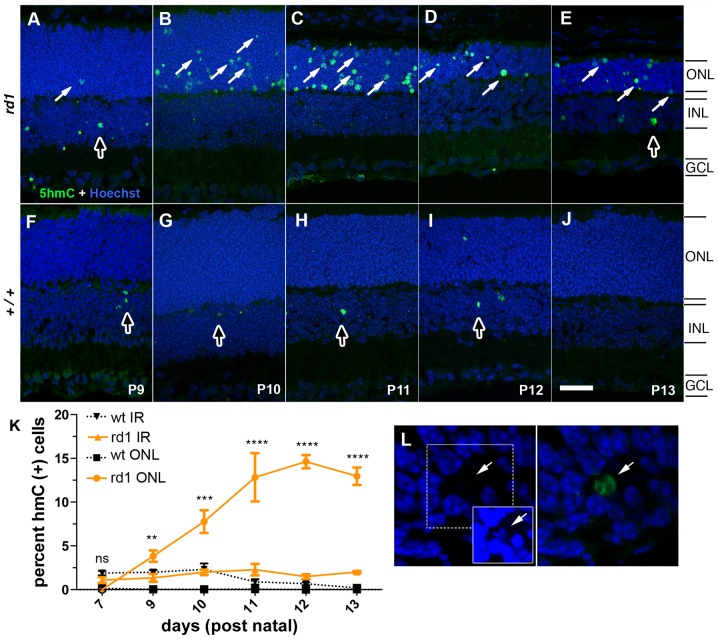
Detection of 5hmC during photoreceptor degeneration in the *rd1* mouse. (A–E) *Rd1* or (F–J) wild-type mouse retinas, P9 through P13, were labeled with antibodies against 5hmC (A–J). White arrows indicate 5hmC (+) cells in the ONL, dark arrows indicate occasional (+) cells in the INL. (K) Quantification of percent positive cells in the outer or inner retina for wild-type (+/+) and *rd1* are plotted as a function of days of age. (L) 5hmC (+) cells in the ONL with decreased staining for intercalating DNA dyes including Hoechst. The inset is the same image taken at a significantly higher gain. Black squares = +/+ ONL, black triangles = +/+ inner retina, orange circle = *rd1* ONL, orange triangle = *rd1* inner retina. Values are mean ± sem. ns = not significant, ****p<0.0001 versus wild type. ONL = outer nuclear layer; INL = inner nuclear layer; GCL = ganglion cell layer. Scale bar (A) = 25 µM.

Images of tissue sections were acquired with a Zeiss Axioplan2, Zeiss LSM510 or LSM710 laser scanning confocal microscope. Confocal microscopy was performed with similar settings for laser power, photomultiplier gain and offset, with a pinhole diameter of one Airey unit. In most cases, thin optical sections (∼0.5–1 µm) were used to study subcellular localization or co-localization (Figures 1, 2, 3, 4, 5, and 6). When quantitation was not required, images were adjusted for brightness and contrast using Aperture (Apple Computer Inc.), ImageJ (NIH; http://rsb.info.nih.gov/ij/) or Adobe Photoshop to in order to normalize images for subtle variation in background fluorescence or to highlight certain aspects of morphology (e.g Figure 7L). Maximum intensity projection z-stacks (5–10 optical sections, 0.5–1.0 µm thickness, 0.3 µm step size) were rendered to give a more inclusive picture within the tissue sections (Figure 7).

### Quantitation

The number of 5hmC (+) cells in adjacent fundal regions of the retina from each time point for wild-type and *rd1* were counted for each age group (n ≥7). For comparison of cell numbers within a given experiment, digital images taken from each group were acquired with a similar digital gain. Brightness and contrast settings were not altered in datasets involving quantitation. 5hmC (+) cells from each age group were manually counted while the total number of Hoechst (+) cells was measured by automated particle analysis with ImageJ using ITCNv1.6 (Image-based Tool for Counting Nuclei; width 19 pixels; minimum distance 9.5 pixels). The number of positive cells was divided by the total number of cells and expressed as a percentage of positive cells per retinal layer. By sampling small regions of the retina with 5hmC (+) cells and comparing that manually to the nuclear counter stain, we discovered that the ITCN quantitation algorithm used to detect and count total cell numbers gave a small, but significant, under representation of the total cell count. This was largely attributable to differences between signal intensities between the lightly stained Hoechst signals in 5hmC (+) cells and the brightly stained 5hmC (−) cells. No automated method of analysis that we tested gave a completely accurate total cell count, so to compensate we deducted the number of 5hmC positive cells from the total cell number and used the following equation to deduce the percent positive 5hmC cells: N = [H/(T−H)]*100 where N = percent positive cells, H = number 5hmC positive cells counted manually and T = total cell numbers counted by DAPI staining using the ITCN plugin. Cell counts were plotted using GraphPad Prism6 (GraphPad Software, San Diego, CA, USA) with error bars represented as s.e.m. Statistical significance was tested using two-way ANOVA with Bonferroni correction, significance levels were P<0.05 (*), P<0.01 (**), P<0.001 (***) and P<0.0001 (****).

## Results

### The Developing Chicken Embryo Demonstrates Widespread Areas of Hypermethylation

During early embryonic development, numerous regions throughout the chicken embryo stain positive for 5mC (Figure 1). Several regions were decorated with scattered 5mC (+) nuclei (Figure 1B, C, D, E2). The developing brain ventricles, including the optic tectum, showed occasional positive signals (Figure 1B). The posterior located neural tube (nt) and notochord (nc) were largely negative (Figure 1D), although directly adjacent regions were positive. A striking pattern of intensely labeled cells was conspicuously present in the ventral region of the E3 neural retina where a large cluster of cells staining positive for 5mC was present along the morphogenic furrow (Figure 1E2; downward arrow), a region long-recognized to undergo a wave of cell death [14]. 5mC positive cells in the lens were also evident at this time (upward arrow). Regions outside of the brain and eye also contained 5mC reactive structures. For instance, Figure 1D illustrates the many positive cells adjacent to the notochord. A small cluster of 5mC (+) cells along the dorsal aorta was also present (Figure 1D). 5hmC staining patterns in the developing chicken retina were strikingly similar to those of 5mC, particularly within the neural retina and lens (Figure 1F2; arrows). Double labeling with antibodies against 5mC and 5hmC confirmed that these signals colocalized with one another in individual chicken retinal neurons (data not shown). To investigate two additional methylation variants, anti-5fC and anti-5caC antibodies were used but these failed to detect expression of either modified base over a wide dilution range of primary antibody (data not shown).

### The Developing Chicken Retina Demonstrates Focal Areas of Hypermethylation

To assess the extent of DNA methylation during the two waves of cell death known to occur during retinal development, we studied the well-characterized temporal sequence of cell death associated with the developing optic fissure in the chicken at E3–5 (morphogenic PCD) and inner retinal cell death from E7–18 (neurotrophic PCD) [12], [14], [39], [40]. Neurotrophic PCD begins with ganglion cells and progresses to other inner retinal neurons. For these experiments, early (E4), intermediate (E7, 11, and 13) and late (E20) stage embryos covering the period during which both waves of PCD occur in the retina were studied. At E4, when a region specific pattern of morphogenic cell death is occurring, 5mC (+) cells were present along the developing optic fissure (Figure 2A–B). Other positive cells were sporadically found throughout the retina at this time; however, the greatest density was at the ventral aspect of the eye (Figure 2A–B). By E7, when the nuclear layers are laminated and the outer plexiform layer is just beginning to appear, 5mC (+) cells are greatly decreased and are only occasionally seen in the inner retina and generally restricted to the ganglion cell layer (GCL) (Figure 2C). At E11 and 13, when the synaptic plexiform layers are well separated but still maturing, a marked rise in 5mC (+) cells was observed (Figure 2D–E). By comparison, very few cells were found to be 5mC (+) at E20 (Figure 2F) when the major structural aspects of retinal development are complete. At all developmental stages, the PR layer had few to no 5mC (+) cells. In contrast, the vast majority of positive cells during eye development were distributed throughout the inner nuclear layer (INL) and less frequently in the GCL.

### Cleaved Caspase-3 and TUNEL Co-labeling with 5mC in the Developing Chicken Retina

The smaller size and condensed shape of some 5mC (+) cells suggested that they might represent a later stage of degeneration in the apoptotic pathway. To test this hypothesis, we performed simultaneous labeling for 5mC and either active cCaspase-3, an early stage marker of apoptosis, or TUNEL, a marker of late cell death, including but not restricted to apoptotic cells. Double labeling at E11 for either anti-cCaspase-3 or TUNEL and 5mC demonstrated more cCaspase-3 (+) and TUNEL (+) cells than 5mC (+) cells (Figure 3); however, while there was relatively little overlap between cCaspase-3 (+) and 5mC (+) cells (Figure 3A–E), there was a high degree of overlap between the TUNEL (+) and 5mC (+) cells (Figure 3F–H) suggesting that 5mC (+) cells are either in a later stage of apoptosis or are possibly involved in a TUNEL (+) caspase-independent pathway. Tissue sections from earlier E3 chick retinas and non-retinal tissues elsewhere in the embryo also showed similar staining patterns (Figure S2).

### Degenerating *rd1* Retinas Demonstrate Focal Areas of Hypermethylation

To investigate whether the patterns of 5mC observed in the chicken represent a common biomarker of programmed cell death, we studied the well-characterized *rd1* mouse, which has peak rod degeneration between P12–14. We observed high numbers of 5mC (+) cells in the ONL at P14, near the peak of degeneration (Figure 4A1, B1, D) but not in age-matched wild-type control retinas (Figure 4C). This was observed in both the central (Figure 4A1) and peripheral retina (Figure 4B1), the latter of which had a slightly greater ONL thickness and more 5mC positive cells. This pattern of 5mC staining recapitulates the central to peripheral progression of PR degeneration observed in the *rd1* mouse retina [41], [42]. By P35, only a thin row of PRs remained with occasional 5mC (+) cells present (Figure 4E). Due to the degenerated and rather disorganized state of the PR layer (i.e. lack of outer segments, etc.), definitive identification of cones was difficult. We did see some overlap between Alexa647 conjugated peanut agglutinin lectin (PNAL) and 5mC (+) cells (Figure 4E), suggesting increased 5mC in some cone PRs during cone death.

**Figure 8:**
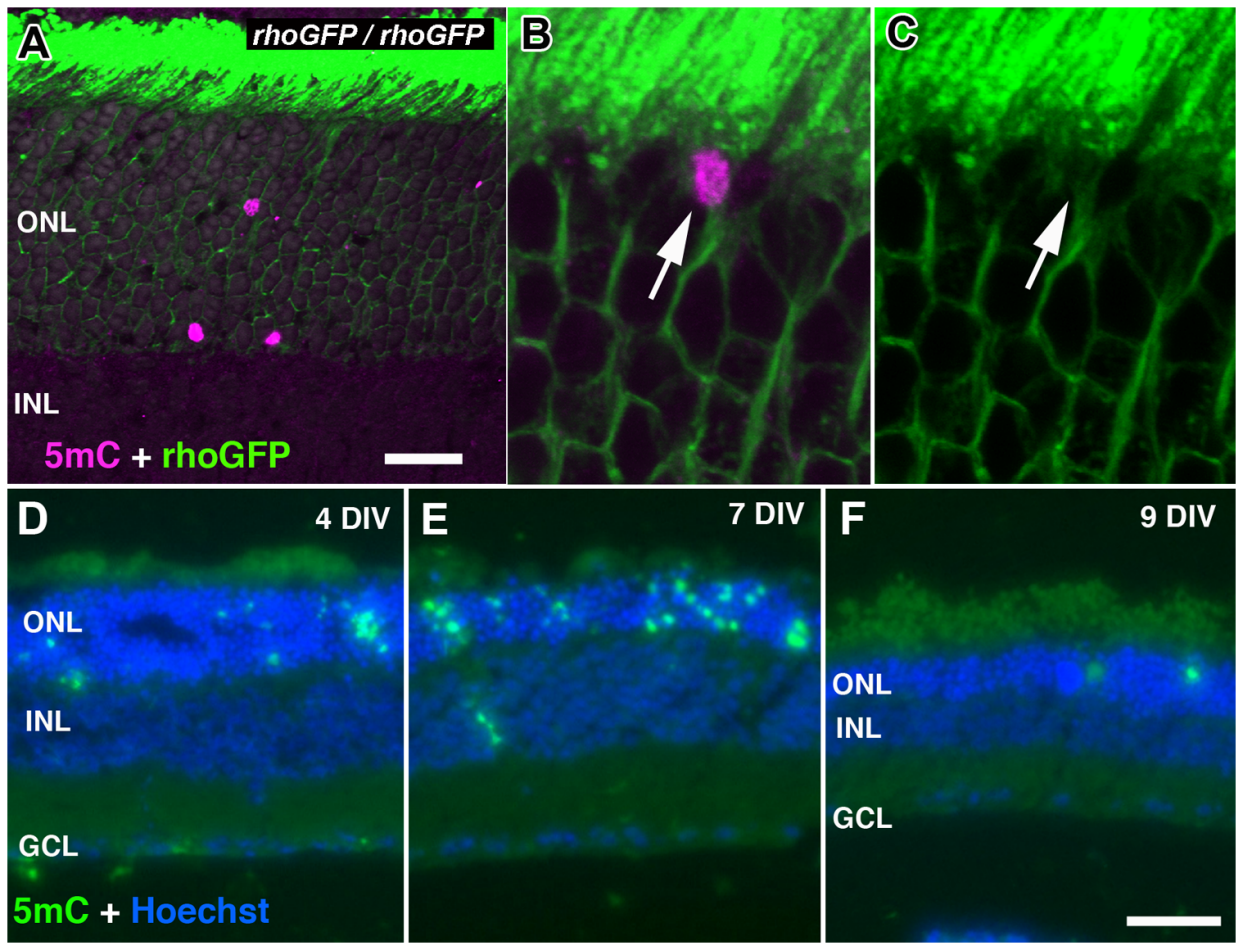
Other retinal degeneration models with elevated 5mC levels. (A) P24 rhoGFP homozygous mice with sporadic PR loss with 5mC staining (magenta) in the ONL (green = GFP in rods). (B–C) Subcellular localization of 5mC (magenta; B) enveloped within a photoreceptor cell body expressing GFP (green; C). (D–F) 5mC staining of a wild type one month-old mouse retina explant undergoing progressive photoreceptor degeneration after 4, 7 and 9 days in vitro (DIV), respectively. ONL = outer nuclear layer; INL = inner nuclear layer; GCL = ganglion cell layer. Scale bars = 20 µM (A) and 100 µM (F).

The shape and size of 5mC (+) PR cells decorating the ONL (Figures 4, 5, 6, and 8) ranged from large normal looking cells with an inverted nuclear appearance to smaller pyknotic cells characteristic of later stages of degeneration [43]. To support the notion that hypermethylated cells belonged to the same population as those undergoing selective cell death as seen in the developing chicken, we performed double labeling with the anti-5mC antibody and TUNEL at P14 (Figure 5A–F). Like the developing chicken retina (Figure 3), more cells were TUNEL (+) than 5mC (+), while most 5mC (+) cells were TUNEL (+). Interestingly, we saw the same staining pattern for 5hmC (Figure 5G–I) leading us to speculate that the 5mC and 5hmC antibodies were labeling the same cells. When P11 mouse retinas were probed with antibodies against both 5mC and 5hmC, a high degree of colocalization was observed indicating that most if not all 5mC (+) cells were also 5hmC (+) (Figure S1G–I). While detection of TUNEL positive cells can be an indication of apoptosis, cells dying by non-apoptotic mechanisms can also be TUNEL positive [44]. We therefore investigated whether there was any overlap between 5mC and cleaved caspase-3, which is considered a more specific marker of apoptosis. Several previous reports have failed to detect cleaved caspase-3 in retinal degeneration models including the *rd1* mouse [18], [34]. Our analysis confirmed these findings in that we failed to detect cleaved caspase-3 in the outer nuclear layer of *rd1* retinas undergoing degeneration (Figures 5J–L and Figure S4). This finding is consistent with a caspase-independent form of cell death. During periods of developmental cell loss (P4–P10) both 5mC signals and cCaspase-3 were evident within the inner retina; however, each had similar yet distinct patterns of cell labeling (Figure S4). These distinct patterns were similar to what was observed during development of the chicken nervous system in that the majority of 5mC and cCasp3 signals did not overlap. Since cCaspase-3 did not co-label with 5mC in the ONL of degenerating retinas, we expanded our analysis of cell death markers to poly ADP-ribose (PAR)(Figure 6). At both P10 and P14 we observed that many, but not all, 5mC (+) cells in the outer nuclear layer of degenerate animals exhibited PAR accumulation as compared to wild type controls, which exhibited no such staining. Interestingly, although many cells showed overlap between 5mC and PAR signals (solid white arrows), not all cells expressed both markers; the outer retina (Figure 6B and C; upward arrows) and inner retina (Figure 6E and F; downward arrows) each contained cells that were positive for 5mC but not PAR.

### Time Course of 5mC and 5hmC Staining in the *rd1* Retina

Retinal degeneration in the *rd1* mouse is characterized by a rapid death of rods between P10 and P15, resulting in a thinning of the ONL. After 21 days, only 1–2 rows of cone PRs remain [16]. To determine the temporal pattern of methylation and hydroxymethylation in the degenerating retina prior to, during and after rod degeneration, a time course from P7–14 and P35 was examined in retinal tissue sections from *rd1* and wild-type mice with 5mC (Figures 4 and Figure S3) and/or 5hmC (Figure 7) specific antibodies. At P7, no 5mC (+) or 5hmC (+) cells were seen in the ONL of either the wild-type or *rd1* mice (data not shown). We saw occasional 5hmC (+) signals in the inner retinas of wild-type and *rd1* mice at all stages of degeneration (Figure 7A–J). Antibodies against 5mC (Figure S3) demonstrated a nearly identical labeling pattern as 5hmC antibodies. Although still scarce at this stage, we saw 5hmC (+) PRs as early as P9 (Figure 7A). By P10, before the ONL had become noticeably thinner, the number of 5hmC cells increased dramatically (Figure 7B,K). As the ONL thinned as a result of cell loss, the number of 5hmC (+) cells gradually decreased (Figure 7C–E). With the exception of a few scarce immunoreactive cells in the inner retina, wild-type control retinas at all stages each had an outer nuclear layer that was consistently negative for 5mC or 5hmC (Figure 7F–J) which is reminiscent of the TUNEL staining patterns characteristic of normal developmental cell loss. Throughout these experiments, we observed that cells containing advanced stages of cell death were often weakly stained for Hoechst even though they were 5mC (+) or 5hmC (+) (Figure 7L). By increasing the gain of digital images, Hoechst signals that were initially too weak to see were identifiable (Figure 7L; inset panel).

### Hypermethylation in Other Models of PR Cell Death

To explore whether changes in 5mC are unique to the *rd1* mouse or part of a broader cell death process, we inspected the staining pattern of 5mC in homozygous rhodopsin-GFP knock-in mice (*hrGFP)* that are known to undergo a similar, albeit slower, PR degeneration beginning around 1 month after birth [23]. In P24 mice, at a relatively early stage of degeneration, 5mC (+) cells were found to be present in the ONL (Figure 8A–C). We also studied a short time course of adult retinal explant tissues grown for 4, 7 and 9 days in vitro (DIV) since we have previously seen that this period is accompanied by a rapid degeneration of the outer retina that includes TUNEL (+) cells (unpublished observation). In addition to the steady progression of cell loss, resulting in a considerably thinner ONL by 9 DIV, we observed 5mC (+) cells beginning to accumulate in the ONL at 4 DIV (Figure 8D). At 7 DIV, the number of 5mC (+) cells was higher in the noticeably thinner ONL (Figure 8E). By 9 DIV, when the ONL was even thinner, the number of 5mC (+) cells was diminished (Figure 8F).

## Discussion

Dynamic regulation of genomic DNA methylation is critical for vertebrate development and has been linked to cellular processes such as regulation of gene expression, suppression of parasitic repetitive elements, and carcinogenesis [45]. In the current study, we used immunohistochemical staining against 5mC and 5hmC to demonstrate a novel link between epigenetic modifications to DNA and programmed cell death of retinal neurons during normal retinal development and PR degeneration. These results can be summarized as follows: 1) During the course of normal retinal development, apoptotic cell loss within the inner retina was accompanied by increased detection of 5mC and 5hmC. These epigenetic marks showed greater overlap with late (e.g TUNEL) as opposed to early cell death markers (cCaspase-3). 2) Increased detection of 5mC and 5hmC also correlated with late (e.g TUNEL or PAR) but not early cell death markers (e.g. cCaspase-3) in PRs in several models of retinal degeneration. 3) 5mC (+) and 5hmC (+) PRs were always TUNEL (+); however, the converse was not true as there were often TUNEL (+) cells that were not 5mC (+) or 5hmC (+). 4) 5mC positive cells of the *rd1* mouse retina ONL were not co-labeled with cCaspase-3 but were co-labeled with antibodies against PAR. Collectively these results suggest a previously unrecognized role for DNA methylation during programmed cell death in retinal neurons.

In the past, DNA fragmentation and nuclear condensation, cardinal features of apoptosis, categorized the *rd1* mouse as a typical model for retinal apoptosis [46]. However, as molecular insights into pathways involving caspases, Poly(ADP-Ribose) Polymerase (PARP), CaMKII (calmodulin kinase II), calpain and other mediators of cell death have been uncovered, this view has evolved [35], [47]–[49]. Initially, Kim et al showed by Western blot and immunohistochemistry that the *rd1* mouse exhibits a significant increase in cleaved caspase-3 around the peak of apoptotic cell death [21]. Yoshizawa et al [22] further demonstrated that a caspase-3 inhibitor could transiently delay inherited retinal degeneration in C3H mice carrying the *rd1* allele, a finding supported by a subsequent study showing that *rd1*/caspase-3 double mutant mice have a delay in photoreceptor loss, albeit a temporary one [19]. Since small molecules can be promiscuous and transgenic animals may have an altered course of development, it can be difficult to distinguish whether effects on the capase pathway are direct or indirect. An ER stress model involving the mitochondrion and endoplasmic reticulum in degenerating photoreceptors is supported by two studies demonstrating that AIF and caspase-12 are translocated to the nucleus in *rd1* photoreceptors [50], [51]. In a more recent study that contradicts some earlier findings, Sanges et al, were unable to detect cleaved caspase-3 in extracts from wild-type or *rd1* animals. Other evidence in favor a caspase-independent cell death in the *rd1* mouse comes from reports that degeneration is independent of caspase-9, -8, -7, -3, and -2 activation [18], [20]. It is difficult to dismiss outright the earlier finding of cleaved caspase-3 in degenerating retinas; however, since our own analysis failed to uncover caspase-3 activation in the outer retina, even when we saw considerable activation in the inner retina, we tend to favor the hypothesis that photoreceptor cell death in the *rd1* mouse occurs by ‘parthanatos’ or a caspase-independent mechanism caused by activation of an intrinsic cell death program due to excessive activation of PARP [52], [53]. Given the conflicting nature of some of these studies, it is clear that a more in-depth analysis of the *rd1* mutation in various mouse strains could be extremely helpful as would a comparative analysis of other retinal degeneration mouse models similar to what has been done with P23H and S334ter mutant rats [34].

Several studies have linked dynamic changes in DNA methylation to programmed cell death in neurons and neuronal support cells. In murine motor neurons, upregulation of Dnmt and increased DNA methylation led to apoptosis. Conversely, inhibition of Dnmt activity prevented increased accumulation of 5mC and apoptosis of motor neurons [54]. A separate study using cultured astrocytes demonstrated that disruption of Dnmt1 activity resulted in global hypomethylation of the genome. These hypomethylated astrocytes adopted characteristics of gliomas including increased proliferation and evasion of PCD in a chemically-induced model of apoptosis [55]. If a similar reversible relationship exists for retinal cell death, these observations in other types of neurons may be highly relevant to addressing retinal disease and the development of new therapeutic strategies. If on the other hand, the accumulation of these hypermethylated marks turns out to be a biomarker for late-stage disease, then it is unlikely that strategies aimed at rescuing dying through suppression of DNA methylation will be successful. Attempts to rescue dying cells by altering DNA methylation, specifically though suppression of Dnmts, will be very useful for addressing this issue.

DNA methylation is known to have an important role in the development, differentiation, and homeostasis of retinal neurons [10], [11], [56]. Dnmts are expressed in mouse retinal progenitors as well as in mature retinal neurons and are likely required for establishing normal cell-specific patterns of DNA methylation in the retina [6], [9], [57]. Our study has identified dynamic regulation of DNA methylation that occurs in concert with programmed cell death of retinal neurons. These results suggest that while the genetic targets of this dynamic regulation have yet to be identified, DNA methylation may play a vital role in retinal cell death.

Striking was the increased detection of both 5mC and 5hmC staining during the loss of retinal neurons. Recently, Tet enzymes have been shown to oxidize 5mC to 5hmC, which is hypothesized to be an early intermediate in an active DNA demethylation pathway [3]. Interestingly, 5hmC is particularly abundant in neurons [58], [59]. A balance between DNA methylation and hydroxymethylation is likely required for homeostasis in post-mitotic neurons. The presence of both 5mC and 5hmC during retinal cell death suggests a complex process involving both active methylation and demethylation pathways.

DNA methylation is one of several epigenetic modifications known to recruit repressive chromatin complexes to genomic loci. An important role for another epigenetic mechanism, deacetylation of histones, has been demonstrated in two mouse models of retinal neurodegeneration. Increased HDAC activity was found in *rd1* PRs undergoing programmed cell death, and mutant PRs could be protected by *in vivo* treatment with HDAC inhibitors [60]. A similar mechanism of HDAC induction was seen in murine retinal ganglion cells (RGCs) following induced axonal injury. Systemic delivery of HDAC inhibitors was shown to reduce RGC death after axon injury [61]. Our study demonstrates dynamic regulation of DNA methylation prior to programmed cell death of retinal neurons. An interesting notion that arises from this study is the potential for cross talk between epigenetic signals during programmed cell death. Since a synergistic effect between inhibitors of histone deacetylation and DNA methylation has been observed in cancer cells [62], [63], it is quite possible that demethylation combined with HDAC inhibition may lead to even greater neuroprotection in models of retinopathy.

In summary, methylation and hydroxymethylation of genomic DNA appear to be important mechanisms that participate in the programmed cell death of retinal neurons. The full extent of these novel mechanisms remains to be determined. Future studies investigating genomic targets of 5mC and 5hmC during programmed cell death and the specific enzymes responsible for these modifications will be useful in further characterizing this pathway. We are hopeful that a better understanding of the relationship between epigenetic modifications and retinal cell death will offer new insights into normal retinal development and pathways leading to retinal degeneration.

## Supporting Information

Figure S1 **Specificity of antibodies used to probe DNA methylation.** (A) A ∼450 bp amplicon made with either NTP’s containing unlabeled cytosine (lane1), 5mC (lane2), or 5hmC (lane3) containing dNTP mixes and digested with *Bso*BI, a methyl sensitive restriction enzyme, to verify incorporation of methyl group modified PCR product. (B–C) Modified amplicons were spotted onto nylon membranes and probed with antibodies against (B) 5hmC or (C) 5mC. Columns 1–3 of the dot blot are the same as indicated in Panel A. (D–F) Tissue sections from P11 *rd1* retinas stained with (D) 5hmC or (E and F) 5mC antibodies pre-adsorbed with unmodified or modified PCR products. (G–I) Tissue section from a P11 *rd1* retina co-stained with 5mC (G) and 5hmC (H). The merged image (I) illustrates the high degree of overlap between these signals.
(TIF)

Figure S2 **Comparison of 5mC and cleaved caspase-3 in the early E3 chick embyro.** (A–C) chick retinas around the optic fissure labeled with cCaspase3 or 5mC. Arrows indicate areas of overlap while inverted dark arrows indicated non-overlapping signals. (D–F) 5mC and cCaspase3 in other non-retinal embryonic structures.
(TIF)

Figure S3 **5mC staining of photoreceptor degeneration in the** ***rd1*** **mouse.** (A–E) 5mC staining of the *rd1* mouse retina from P9–P13. Dark arrows indicate 5mC (+) cells in the inner nuclear layer while white arrows indicate positive cells in the outer nuclear layer. ONL = outer nuclear layer; INL = inner nuclear layer; GCL = ganglion cell layer.
(TIF)

Figure S4 **cCaspase-3 and 5mC in the developing and degenerate mouse retina.** Retinal sections were co-labeled in a developmental series ranging in age from P4 (A–C), P6 (D–F), P10 (G–L), and P14 (M–R). Wild type control sections (A–I, M–O) generally exhibited a high degree of separation in the inner retina, while *rd1* retinas (J–L, P–R) showed additional staining in the ONL for 5mC but not cCaspase3. ONL = outer nuclear layer; INL = inner nuclear layer; GCL = ganglion cell layer.
(TIF)
