# Supplementary figures and images for: Epigenetics and Cell Death: DNA Hypermethylation in Programmed Retinal Cell Death
Source: PLoS One. 2013 Nov 11;8(11):e79140. doi: 10.1371/journal.pone.0079140 (PMC3823652; doi:10.1371/journal.pone.0079140)

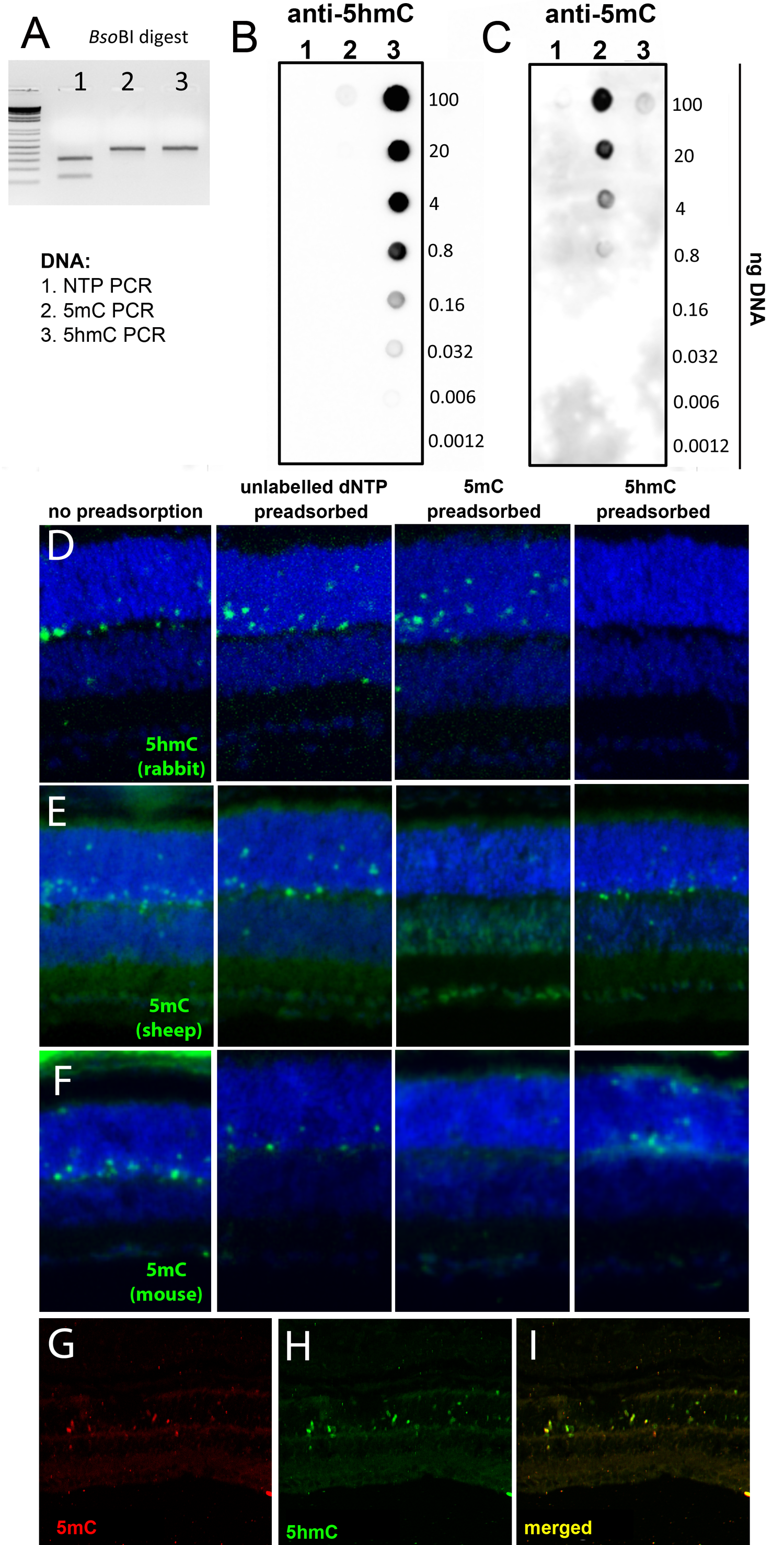

Supplement: Figure S1 — Specificity of antibodies used to probe DNA methylation. (A) A ∼450 bp amplicon made with either NTP’s containing unlabeled cytosine (lane1), 5mC (lane2), or 5hmC (lane3) containing dNTP mixes and digested with BsoBI, a methyl sensitive restriction enzyme, to verify incorporation of methyl group modified PCR product. (B–C) Modified amplicons were spotted onto nylon membranes and probed with antibodies against (B) 5hmC or (C) 5mC. Columns 1–3 of the dot blot are the same as indicated in Panel A. (D–F) Tissue sections from P11 rd1 retinas stained with (D) 5hmC or (E and F) 5mC antibodies pre-adsorbed with unmodified or modified PCR products. (G–I) Tissue section from a P11 rd1 retina co-stained with 5mC (G) and 5hmC (H). The merged image (I) illustrates the high degree of overlap between these signals. (TIF) [file pone.0079140.s001.tif]

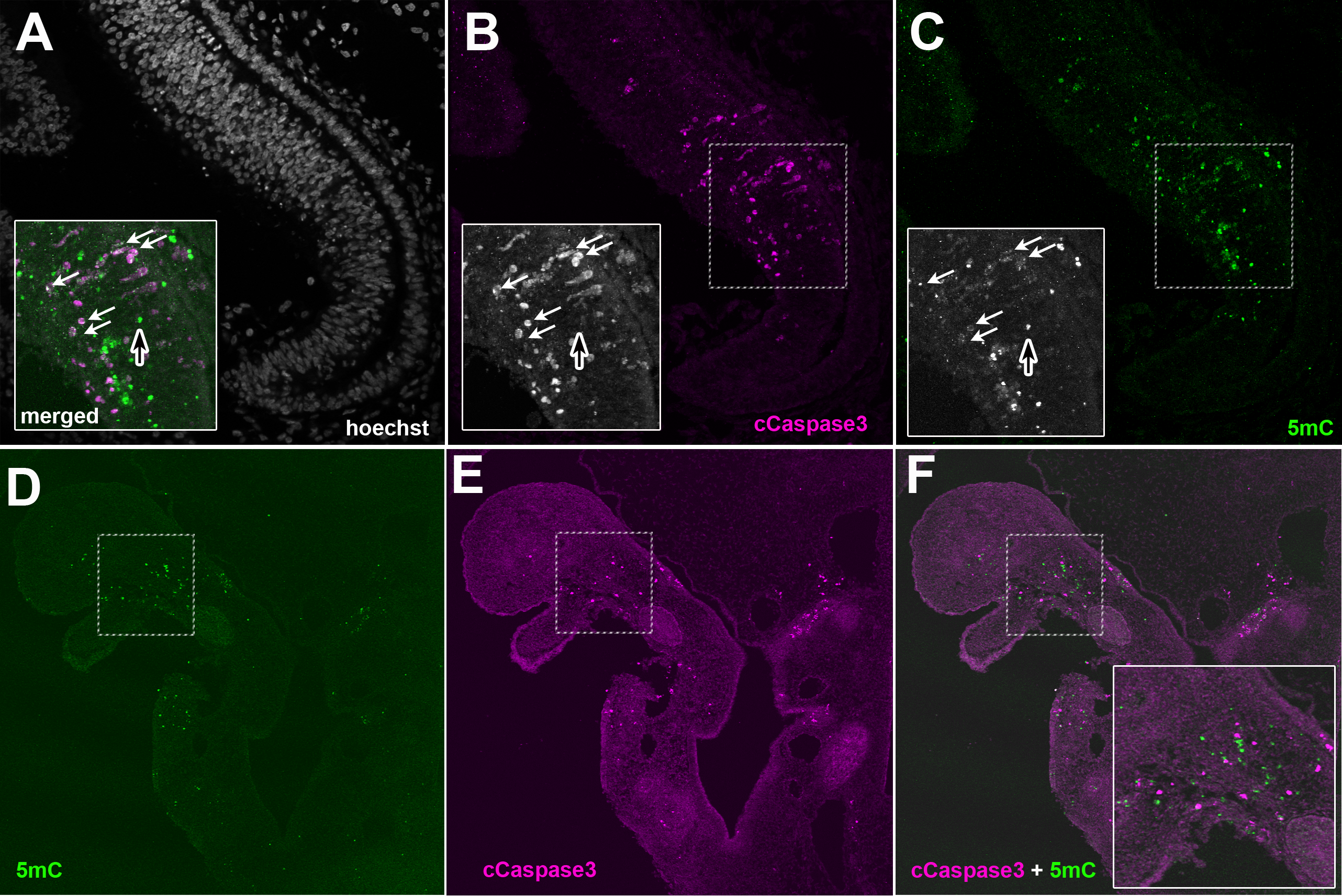

Supplement: Figure S2 — Comparison of 5mC and cleaved caspase-3 in the early E3 chick embyro. (A–C) chick retinas around the optic fissure labeled with cCaspase3 or 5mC. Arrows indicate areas of overlap while inverted dark arrows indicated non-overlapping signals. (D–F) 5mC and cCaspase3 in other non-retinal embryonic structures. (TIF) [file pone.0079140.s002.tif]

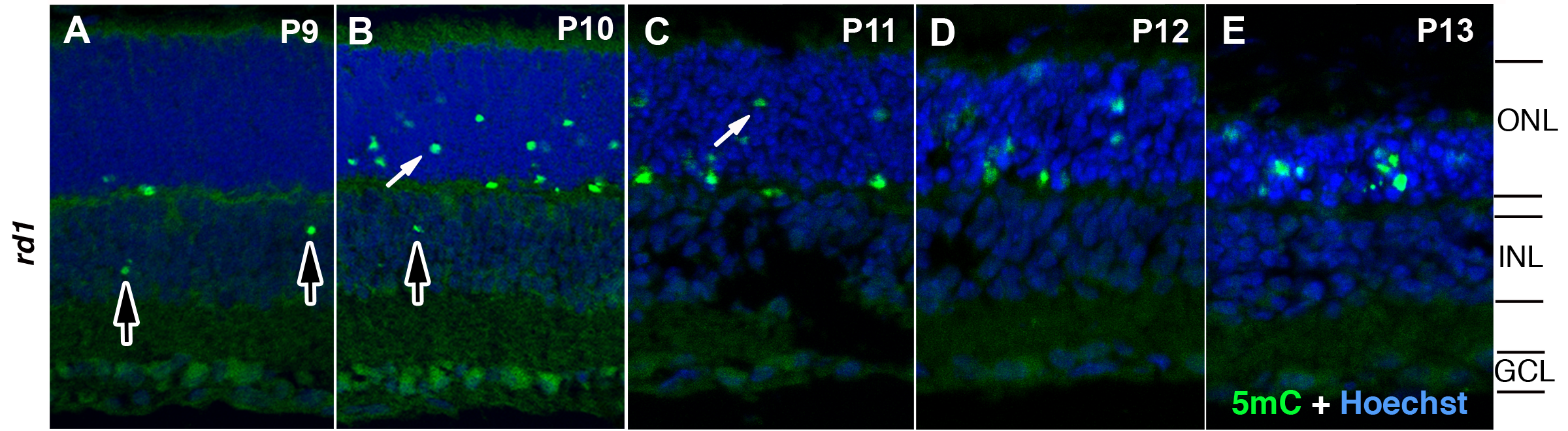

Supplement: Figure S3 — 5mC staining of photoreceptor degeneration in the rd1 mouse. (A–E) 5mC staining of the rd1 mouse retina from P9–P13. Dark arrows indicate 5mC (+) cells in the inner nuclear layer while white arrows indicate positive cells in the outer nuclear layer. ONL = outer nuclear layer; INL = inner nuclear layer; GCL = ganglion cell layer. (TIF) [file pone.0079140.s003.tif]

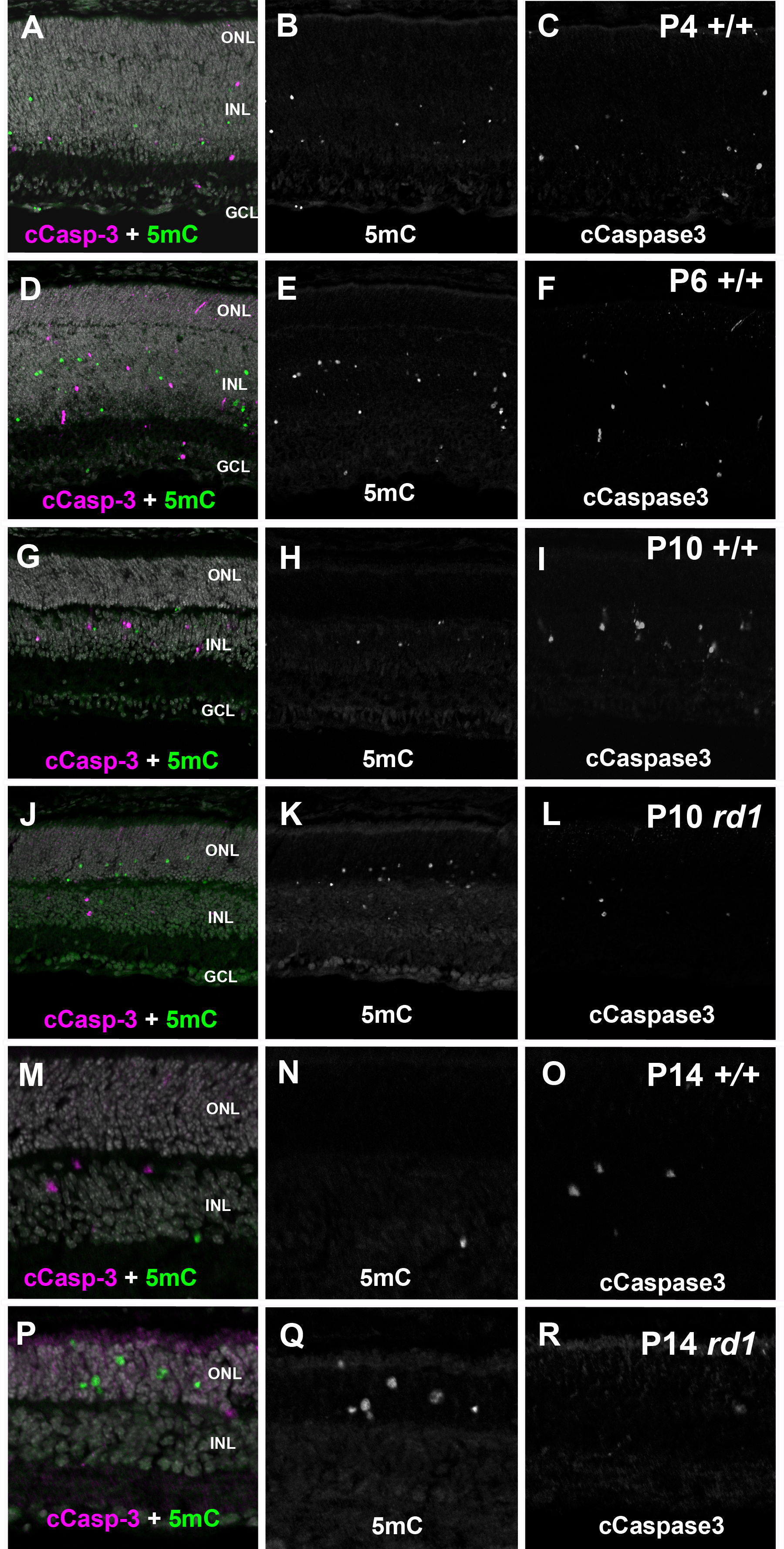

Supplement: Figure S4 — cCaspase-3 and 5mC in the developing and degenerate mouse retina. Retinal sections were co-labeled in a developmental series ranging in age from P4 (A–C), P6 (D–F), P10 (G–L), and P14 (M–R). Wild type control sections (A–I, M–O) generally exhibited a high degree of separation in the inner retina, while rd1 retinas (J–L, P–R) showed additional staining in the ONL for 5mC but not cCaspase3. ONL = outer nuclear layer; INL = inner nuclear layer; GCL = ganglion cell layer. (TIF) [file pone.0079140.s004.tif]
